# HDL Functionality, Cholesterol-Related Biomarkers and Incidence of Dementia: A Case–Cohort Study in Older Adults at High Cardiovascular Risk

**DOI:** 10.3390/ijms27209197

**Published:** 2026-10-16

**Authors:** Mireia Malcampo, Álvaro Hernáez, Javier Hernando-Redondo, Emilio Ros, Dolores Corella, Miguel Ángel Martínez-González, Jordi Salas-Salvadó, Ramon Estruch, Juan Timiraos, Enrique Gómez-Gracia, Miquel Fiol, José Lapetra, Lluis Serra-Majem, Antoni Riera-Mestre, Xavier Pintó, Albert Puig-Pijoan, Miguel Ruiz-Canela, José V. Sorlí, Nancy Babio, Rosa Maria Lamuela-Raventós, Olha Khymenets, Aleix Sala-Vila, Olga Castañer, Montserrat Fitó

**Affiliations:** 1 Hospital del Mar Research Institute (IMIM), 08003 Barcelona, Spain; mireiamalcampo@gmail.com (M.M.); ahernaez@researchmar.net (Á.H.); jhernando1@researchmar.net (J.H.-R.); apuig@psmar.cat (A.P.-P.); okhymenets@researchmar.net (O.K.); asala3@researchmar.net (A.S.-V.); 2 Blanquerna School of Health Sciences, University Ramon Llull, 08025 Barcelona, Spain; 3 Consorcio CIBER, M.P. Enfermedades Cardiovasculares (CIBERCV), Instituto de Salud Carlos III, 28029 Madrid, Spain; 4 August Pi i Sunyer Biomedical Research Institute (IDIBAPS), 08036 Barcelona, Spain; erosr@recerca.clinic.cat (E.R.); restruch@clinic.cat (R.E.); 5 CIBER, M.P. Fisiopatología de la Obesidad y Nutrición (CIBEROBN), Instituto de Salud Carlos III, 28029 Madrid, Spain; dolores.corella@uv.es (D.C.); mamartinez@unav.es (M.Á.M.-G.); jordi.salas@urv.cat (J.S.-S.); jjtimiraos@gmail.com (J.T.); mfiolsala@gmail.com (M.F.); joselapetra543@gmail.com (J.L.); lserra@dcc.ulpgc.es (L.S.-M.); ariera@bellvitgehospital.cat (A.R.-M.); xpinto@bellvitgehospital.cat (X.P.); mcanela@unav.es (M.R.-C.); sorli@uv.es (J.V.S.); nancy.babio@urv.cat (N.B.); lamuela@ub.edu (R.M.L.-R.); 6 Department of Preventive Medicine, University of Valencia, 46010 Valencia, Spain; 7 Department of Preventive Medicine and Public Health, Universidad de Navarra, 31008 Pamplona, Spain; 8 Instituto de Investigación Sanitaria de Navarra (IdiSNA), 31008 Pamplona, Spain; 9 Department of Nutrition, Harvard T.H. Chan School of Public Health, Boston, MA 02115, USA; 10 Unitat de Alimentació, Nutrició, Desenvolupament i Salut Mental, Departament de Bioquímica i Biotecnologia, Universitat Rovira i Virgili, 43201 Reus, Spain; 11 Institut d’Investigació Sanitària Pere Virgili (IISPV), 43204 Reus, Spain; 12 Internal Medicine Service, Hospital Clínic, 08036 Barcelona, Spain; 13 Department of Cardiology, Hospital Universitario de Álava, 01004 Vitoria, Spain; 14 Department of Preventive Medicine, University of Málaga, 29071 Malaga, Spain; gomez_gracia@uma.es; 15 Research Group on Nutritional Epidemiology & Cardiovascular Physiopathology, Health Research Institute of the Balearic Islands, University Hospital Son Espases, 07120 Palma de Mallorca, Spain; 16 Research Unit, Department of Family Medicine, Distrito Sanitario Atención Primaria Sevilla, 41013 Sevilla, Spain; 17 Research Group in Nutritional Epidemiology and Cardiovascular Pathophysiology, Instituto de Investigación Sanitaria Illes Balears (IdISBa), 35016 Las Palmas de Gran Canarias, Spain; 18 Servicio Canario de Salud, Centro Hospitalario Universitario Insular Materno Infantil, 35016 Las Palmas de Gran Canarias, Spain; 19 Lipids and Vascular Risk Unit, Internal Medicine, Hospital Universitario de Bellvitge Institut d’Investigació Biomèdica de Bellvitge, 08907 Hospitalet de Llobregat, Spain; 20 Faculty of Medicine and Health Sciences, Universitat de Barcelona, 08036 Barcelona, Spain; 21 Cognitive and Behavioural Neurology Unit, Department of Neurology, Hospital del Mar, 08003 Barcelona, Spain; 22 Polyphenol Research Group, Department of Nutrition, Food Science and Gastronomy, Faculty of Pharmacy and Food Sciences, INSA-UB, University of Barcelona, 08921 Barcelona, Spain; 23 CIBER, M.P. Epidemiología y Salud Pública (CIBERESP), Instituto de Salud Carlos III, 28029 Madrid, Spain; 24 Primary Care Research Unit Metropolitana Sud, Institut Català de la Salut, 08820 El Prat de Llobregat, Spain; 25 Institut Universitari de Recerca en Atenció Primària Jordi Gol i Gurina, 08007 Barcelona, Spain

**Keywords:** high-density lipoprotein, lipid, cholesterol efflux capacity, cardiovascular, neurodegenerative disease, dementia, Alzheimer’s disease

## Abstract

Associations of lipid profiles, including qualitative and functional high-density lipoprotein (HDL) traits, and cholesterol-related biomarkers with incident dementia and Alzheimer’s disease (AD), remain unclear. In this regard, older adults at high cardiovascular risk are at particularly high risk of developing dementia. The aim is to evaluate whether these biomarkers are associated with the incidence of dementia and AD in older adults at high cardiovascular (CV) risk. In a case–cohort study, we selected 106 incident dementia cases (82 AD) and a random subcohort of 201 non-cases. We quantified (baseline and at one-year), lipid profile; HDL-mediate cholesterol efflux capacity in a model of human neurons; HDL-mediated anti-inflammatory protection in a model of brain endothelial cells; 24S-hydroxycholesterol and apolipoprotein E. Multivariable-adjusted Cox models were addressed. HDL cholesterol was inversely associated, whereas triglycerides directly associated, with dementia and AD incidence. Higher HDL-mediated cholesterol efflux was inversely associated with dementia (HR 0.64, 95% confidence interval (CI) 0.45–0.91) and AD (HR 0.52, 0.35–0.76). Greater HDL-mediated endothelial protection was associated with lower dementia and AD incidence (HR 0.68, 95% CI 0.47 to 0.99 and HR 0.61, 95% CI 0.41 to 0.91, respectively). A one-year increase in 24S-hydroxycholesterol was associated with a higher incidence of any dementia (HR 2.38, 1.33–4.27) and AD (HR 2.24, 1.11–4.53). Enhanced HDL functional properties ex vivo, in preclinical models using human brain cells, together with lower levels of 24S-hydroxycholesterol, are inversely associated with the incidence of dementia and AD, highlighting these factors as promising prognostic targets in older adults at high CV risk.

## 1. Introduction

With increased life expectancy, the prevalence of chronic age-related degenerative diseases such as cardiovascular (CV) disease (CVD) and dementia has risen. Patients affected by all-cause dementia and Alzheimer’s disease (AD) often exhibit cardiovascular comorbidities such as dyslipidemia, hypertension, type-2 diabetes, and obesity, which may speed cognitive decline. Furthermore, individuals with established CVD or presenting cardiovascular risk factors also show an increased risk of dementia and AD [1]. Thus, older adults at high cardiovascular risk are at particularly high risk of developing dementia. Regarding dyslipidemia, total cholesterol is associated with a higher dementia and AD incidence according to meta-analyses of cohort studies [2,3], particularly for mid-life measurements [3]. Nevertheless, this association is not observed for low-density lipoprotein cholesterol (LDL-C) [2], as supported by Mendelian randomization and genetic studies [4,5]. Meta-analyses of prospective cohort studies have reported a positive association between circulating triglycerides and dementia or AD risk [2]. The association between high-density lipoprotein (HDL) cholesterol (HDL-C) and dementia is not apparent in meta-analyses of prospective studies [2,3], although some prospective studies measuring HDL-C in mid-life have found an inverse association with future incidence of the condition [6] and other studies have found direct associations with the risk of dementia [7,8].

HDL are composed of proteins, lipids, and enzymes that act on endothelial tissue, including the blood–brain barrier (BBB), as multifaceted regulators of BBB homeostasis.

HDLs stabilize tight junctions, reduce vascular leakage, and limit pro-inflammatory molecule entry during systemic inflammation. Functionally, HDL reduce cerebral endothelial inflammation by decreasing adhesion molecules and cytokine production, and limiting leukocyte infiltration. They also provide antioxidant protection by neutralizing free radicals and preventing lipid oxidation, preserving neuronal and endothelial integrity. In addition, HDL promote cholesterol efflux via ABCA1 and ABCG1, remove toxic lipids, maintain membrane fluidity, and support BBB transport and receptor function [9]. Several HDL-like particles have been identified in the human cerebral cerebrospinal fluid [10], which may allow some of its biological functions to occur within the central nervous system. HDL particles display several activities, such as their capacity to remove excess cholesterol (cholesterol efflux capacity), protect vascular cells, exert antioxidant/anti-inflammatory effects, among others [11]. Of note, HDL particles with preserved functionality have been linked to a lower incidence of CVD [12,13], and this role of the HDL function appears to be independent of HDL-C concentrations. Therefore, it is plausible that optimal HDL functional properties may also be associated with a reduced incidence of dementia and AD, independent of HDL-C levels.

The association between apolipoprotein E (ApoE), one of the apolipoproteins present in HDLs, and dementia and AD risk has been widely studied, particularly because carriers of the ε4 allele of the *APOE* gene are at higher risk of AD [14]. ApoE levels have been shown to be lower in the cerebrospinal fluid of AD patients compared with healthy controls [15]. Regarding its connection to CVD, circulating ApoE levels are associated with several cardiovascular risk factors, and Mendelian randomization studies have revealed that genetically determined higher levels of ApoE are linked to a lower risk of coronary and cerebrovascular disease [16]. Nevertheless, the association of circulating ApoE concentrations with the incidence of dementia and AD has not yet been sufficiently studied, and has only been linked to better cognitive function [17]. Finally, cholesterol is pivotal for the central nervous system, because approximately 25% of the body’s cholesterol is located in the human brain [18]. Neuronal degeneration in dementia and AD leads to accumulation of cholesterol in the brain [19], which is exported from the central nervous system into the circulation predominantly as 24S-hydroxycholesterol (24S-HC) [19]. Increased circulating 24S-HC has been shown to be associated with the prevalence of AD, and its use as an early diagnostic marker of AD has been proposed [20].

The aim of this study was to assess whether specific HDL functional properties (HDL capacity to remove cholesterol excess from neuron-like cells and to protect microvascular brain cells) and other cholesterol-related biomarkers (circulating ApoE and 24S-HC levels) are linked to the incidence of dementia and AD in older adults at high cardiovascular risk.

## 2. Results

### 2.1. Study Population

**Figure 1:**
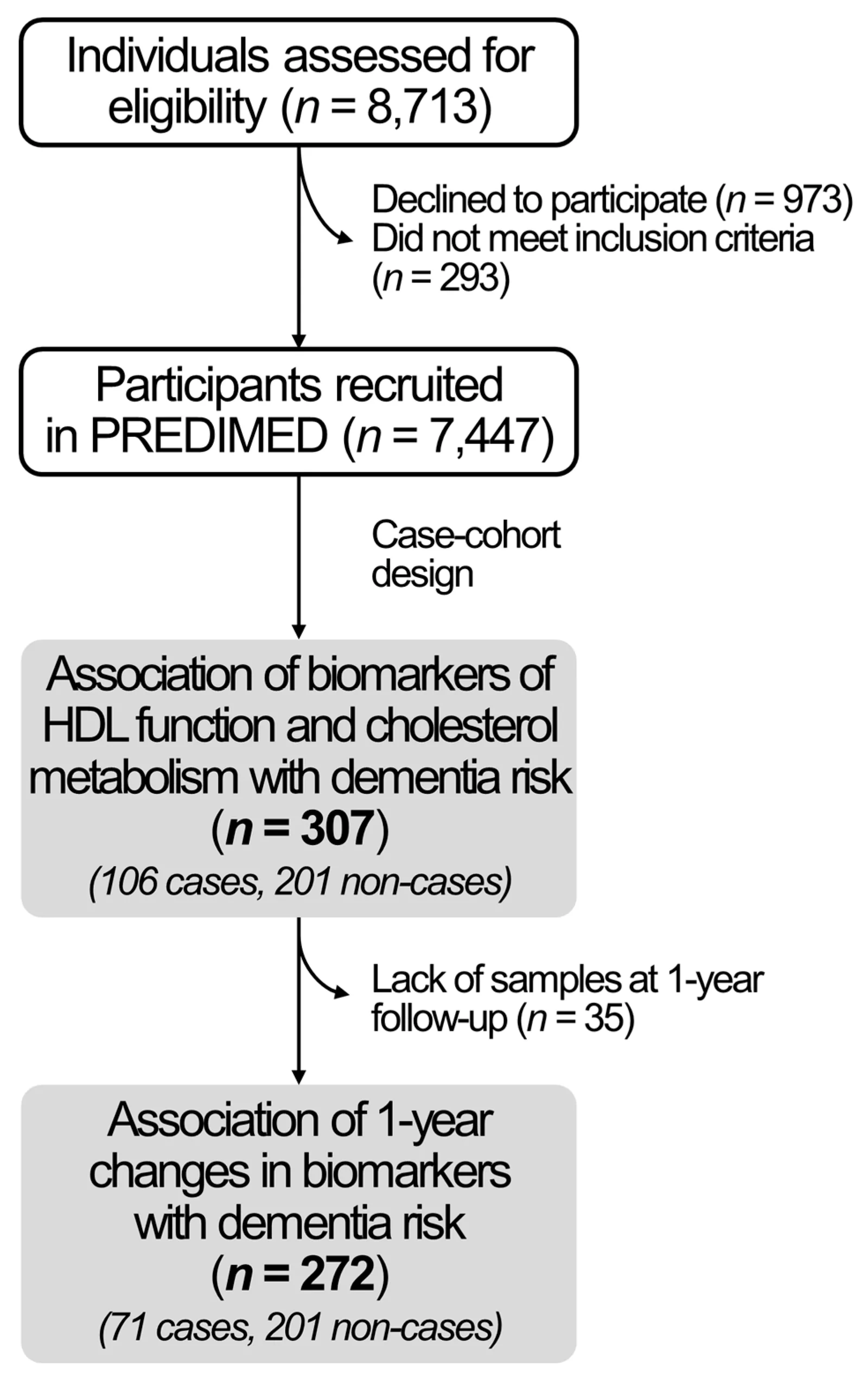
Flow chart.

Flow-chart is described in Figure 1.

Participants with dementia, in comparison with non-cases, tended to be older, had a higher prevalence of diabetes, and were less likely to present with hypercholesterolemia or a history of smoking (Table 1).

Incident AD cases displayed the same pattern and were more frequently women (Supplementary Table S1). An additional analysis comparing the non-AD dementia subgroup with the AD subgroup together with an analysis of the non-AD dementia subgroup has been included as Supplementary material (Supplementary Tables S2 and S3, respectively). Participants with AD had a higher proportion of women compared with non-AD participants.

### 2.2. Lipid Profile and Incidence of Dementia

**Figure 2:**
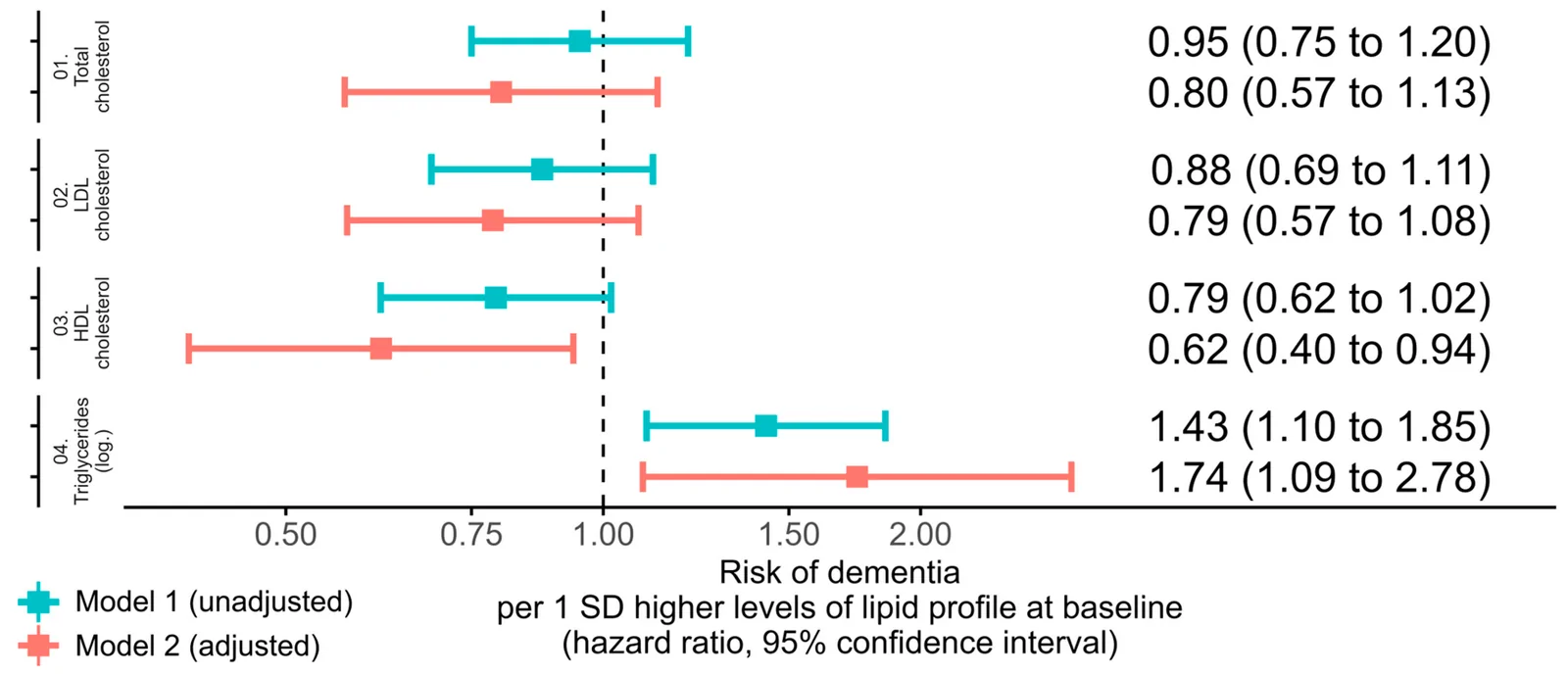
Association of baseline values of lipid profile with incidence of any dementia. Associations between cholesterol-associated biomarkers and the risk of incident dementia were evaluated using the Lin-Ying modification of Cox proportional hazards regression models adapted for the case–cohort design. Results were expressed as hazard ratios per one-standard deviation (SD) increment in cholesterol-associated biomarker levels. Model 1: unadjusted. Model 2: adjusted for age, sex, recruitment site, PREDIMED intervention group, educational level, smoking status, body mass index, *apoE-ε4* genotype, hypertension, diabetes, hypercholesterolemia, and hypertriglyceridemia.

We observed a direct association between circulating triglyceride and dementia incidence, and an inverse association between HDL-C levels and dementia incidence (Figure 2).

Similar patterns were observed when restricting the analysis to AD cases (Supplementary Figure S1). When evaluating one-year changes in lipid profile parameters, a clearer association was found between increases in HDL-C levels and dementia risk, whereas the relationship with triglyceride changes was less precise (Supplementary Figure S2). These trends were similar in the analyses of AD incidence (Supplementary Figure S3).

### 2.3. HDL Functionality and Cholesterol-Related Biomarkers and Incidence of Dementia

**Figure 3:**
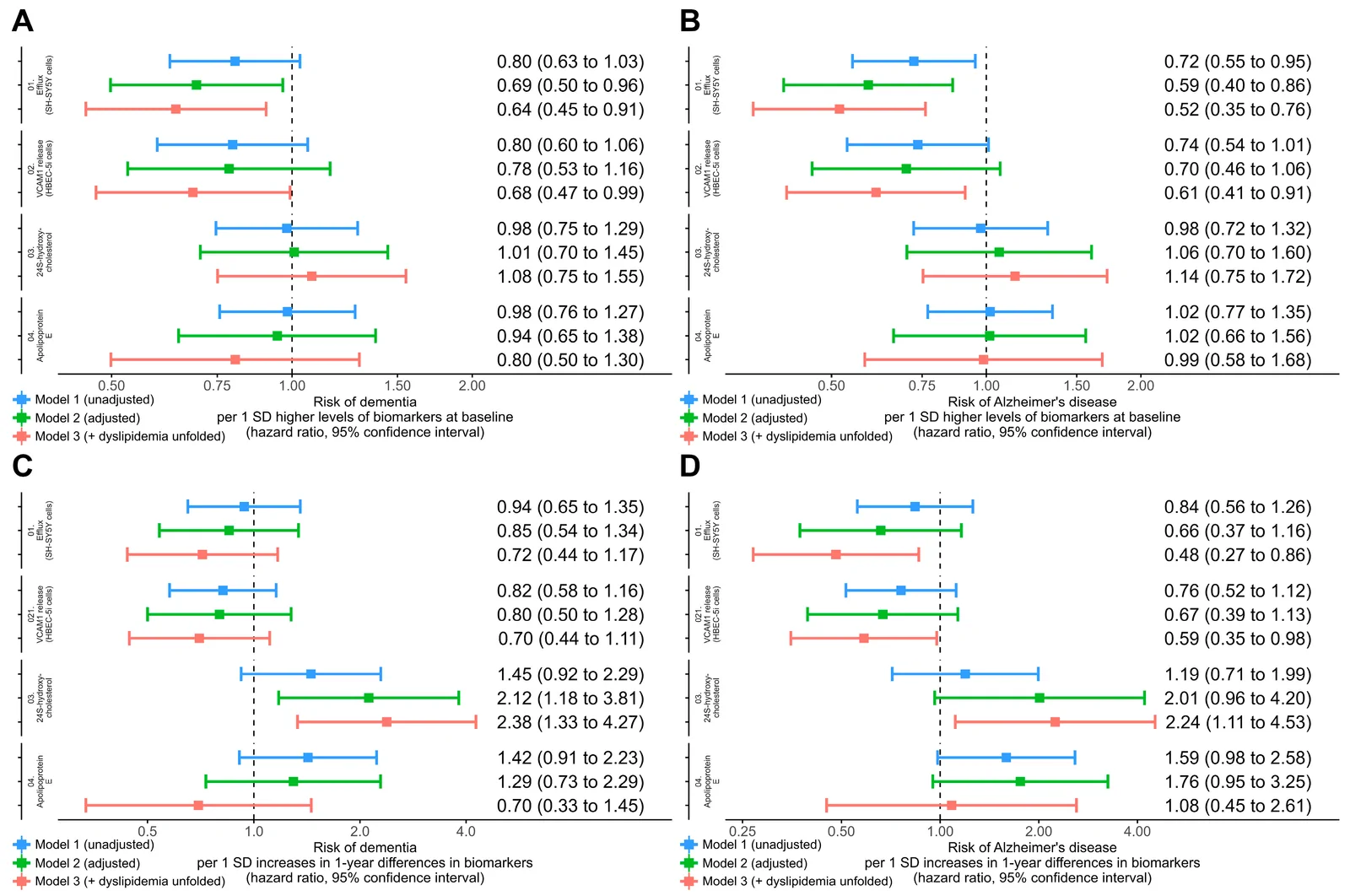
Associations of HDL functionality and cholesterol-associated biomarkers with incidence of dementia at baseline and 1 year after (**A**) all dementias, baseline values; (**B**) AD, baseline values; (**C**) all dementias, one-year differences; (**D**) AD, one-year differences). Associations between cholesterol-associated biomarkers and the risk of incident dementia were evaluated using the Lin-Ying modification of Cox proportional hazards regression models adapted for the case–cohort design. Results were expressed as hazard ratio (HRs) per one-standard deviation (SD) increment in cholesterol-associated biomarker levels. Model 1: unadjusted. Model 2: adjusted for age, sex, recruitment site, PREDIMED intervention group, educational level, smoking status, body mass index, *apoE-ε4* genotype, hypertension, diabetes, hypercholesterolemia, and hypertriglyceridemia. Model 3: adjusted for age, sex, recruitment site, PREDIMED intervention group, educational level, smoking status, body mass index, *apoE-ε4* genotype, hypertension, diabetes, high-density lipoprotein cholesterol (HDL-C), low-density lipoprotein cholesterol (LDL-C), use of cholesterol-lowering medication, triglycerides, and triglyceride-lowering medication. Using the same set of models, we examined the associations between one-year differences in the cholesterol-related biomarkers and dementia incidence (in the subset of individuals with biomarker data after one year of follow-up), as well as the relationships of baseline values and one-year differences in the cholesterol-related biomarkers with the incidence of AD, excluding non-AD dementia cases from these analyses. Cells were seeded at 5 × 10^4^ cells/well in 96-well plates.

An increase in one standard deviation (SD) in HDL cholesterol efflux capacity in human neurons was associated with a 36% lower incidence of dementia (Hazard ratio (HR)Model 3 0.64, 95% CI 0.45 to 0.91; Figure 3A). When we examined the incidence of AD alone, the association was even stronger (HRModel 3 0.52, 95% CI 0.35 to 0.76; Figure 3B), we also observed that an increase in one SD in HDL capacity to protect brain microvascular endothelial cells against inflammatory damage, measured with sVCAM-1 release, was associated with a 32% and 39% lower incidence of incident dementia and AD respectively [HRModel 3 0.68, 95% CI 0.47 to 0.99 (Figure 3A) and HRModel 3 0.61, 95% CI 0.41 to 0.91 (Figure 3B), respectively). The associations of baseline ApoE and 24S-HC levels with incident dementia and AD were near null.

In addition, greater one-year differences in 24S-HC were associated with a higher incidence of any dementia (one-SD increase: HRModel 3 2.38, 95% CI 1.33 to 4.27; Figure 3C) and of AD (one-SD increase: HRModel 3 2.24, 95% CI 1.11 to 4.53; Figure 3C).

A comparison of the absolute values at baseline and at the one-year follow-up has been performed and is included in the supplementary material (Supplementary Table S4). Irrespective of chronic treatment, cholesterol efflux, together with total and LDL cholesterol levels, appears to decrease with ageing.

In the present study population, 24.4% of participants were *APOE4* genotype-positive (16.9% of non-cases and 38.7% of cases; *p* < 0.001). Similarly, within the AD case–cohort, 25.1% of participants were *APOE4* genotype-positive (16.9% of non-cases and 45.1% of cases; *p* < 0.001). Similar results were observed after stratification by *APOE ε4* carrier status (Supplementary Table S5). Among *APOE ε4* -positive carriers, we observed an inverse association between HDL-mediated protection and soluble VCAM-1 release, whereas a direct association was observed with total cholesterol.

Finally, the comparison of baseline demographics, lipid profiles, and dementia risk factors between participants retained in the study and those lost to follow-up is presented in Supplementary Table S6. Older participants and those with diabetes, as well as those assigned to the control group, were more prevalent in the retained group.

## 3. Discussion

Our study examined the associations of HDL functionality and selected cholesterol-related biomarkers with the incidence of dementia and AD in older adults at high cardiovascular risk. Independent of traditional cardiovascular risk factors and dyslipidemia indicators (blood concentrations of HDL-C, LDL-C, and triglycerides, and the use of cholesterol- and triglyceride-lowering medications), we observed inverse associations for the HDL’s capacity to remove excess cholesterol from neurons and its ability to protect brain microvascular endothelial cells from inflammatory damage. In contrast, elevated levels of 24S-HC were associated with a detrimental risk. These associations were more pronounced when analyses were restricted to AD incidence.

Our observations of an inverse association between baseline HDL-C and dementia, together with a direct association for plasma triglycerides agree with some of the previous studies [2,6], suggesting a key role of these lipid profile components in dementia risk. However, our findings also show that HDL functionality, independently of total HDL-C levels, may be more informative than the cholesterol cargo of HDL alone. We found inverse associations between the incidence of dementia and AD and two HDL-mediated actions: cholesterol efflux capacity from experimental neurons and protection of brain microvascular endothelial cells. Lower macrophage-specific cholesterol efflux capacity has been linked to higher risk of subclinical atherosclerosis [21], incident coronary events [13,22,23] and all-cause mortality [24]. However, to the best of our knowledge, this is the first study to demonstrate that HDL functional properties, assessed across two cellular pathways, are associated with a lower risk of dementia, particularly Alzheimer’s disease (AD). Our findings extend these vascular findings into the neurological domain and provide biologically plausible support for the hypothesis that efficient HDL-driven cholesterol mobilization from neurons and HDL-mediated endothelial protection may slow down certain pathological processes that might lead to dementia and AD. HDLs can cross the BBB [10], and bind amyloid-β peptides and excess cholesterol, facilitating their clearance across the BBB [25] and subsequent transport to the liver. An enhanced HDL-mediated endothelial-protection capacity may be linked to a lower likelihood of breakdown of the BBB, which has been postulated as an early indicator of cognitive impairment, dementia and AD [26].

The 24S-HC oxysterol is the primary pathway for eliminating excess cerebral cholesterol; it is generated almost exclusively in neurons by cholesterol 24S-hydroxylase and freely crosses the BBB [27]. Elevated brain (and, by extension, plasma) levels of 24S-HC have been reported to be linked to increased generation of amyloid-β peptides and accelerated neurodegeneration [28]. Clinically, circulating 24S-HC tends to rise in the prodromal and early phases of AD and then decline as neuronal mass decreases [20,27]. One-year changes in 24S-HC were associated with an increased risk of dementia. Nevertheless, we cannot rule out the possibility of reverse causality, as early preclinical neurodegeneration may lead to elevated 24S-HC levels rather than elevated 24S-HC contributing to dementia pathogenesis. Our findings, indicating a direct association between raising 24S-HC concentrations and incidence of dementia and AD, align with this trajectory and support the potential utility of this biomarker in the aging to identify individuals who may subsequently develop dementia and AD.

The relationship between serum ApoE levels and dementia is complex and depends on both circulating ApoE concentrations and the *APOE* genotype. In many observational studies, patients with AD have lower serum ApoE levels than cognitively healthy controls. Individuals carrying the *APOE* ε4 allele, the strongest genetic risk factor for sporadic AD, generally exhibit lower circulating ApoE concentrations than ε3 or ε2 carriers. Reduced ApoE levels may impair the clearance of β-amyloid (Aβ), thereby promoting the accumulation of amyloid plaques in the brain. However, circulating ApoE concentrations do not necessarily reflect ApoE levels within the central nervous system, as the BBB largely restricts the exchange of ApoE between the peripheral circulation and the brain [29].

The main strength of this study is the evaluation of the relationship between cholesterol-related biomarkers and the incidence of all-cause dementia and AD dementia in a well-characterized cohort of older adults at high cardiovascular risk, with rigorous adjustment for potential confounding factors. In addition, we analyzed a comprehensive panel of cholesterol-related biomarkers, including two functional biomarkers derived from preclinical cell-based models.

This work has some limitations. First, AD and related dementias may have a long preclinical phase, often spanning two to three decades from the onset of the first pathological changes in brain to cognitive decline. The clinical data and biological samples in our study were collected at a midpoint in the trajectory, potentially limiting our ability to detect predictors in the early stage if this development. Additionally, we only assessed baseline levels and one-year changes in cholesterol-related biomarkers, which prevented us from examining the associations of long-term biomarkers trajectories with dementia and AD incidence. It should be noted that, in the present study, the findings for all-cause dementia are likely driven predominantly by the AD subgroup. Further studies with earlier and more extended follow-up are needed to confirm these findings. It would also be relevant to determine whether plasma p-tau217 levels, an established diagnostic and prognostic biomarker, correlate with HDL function, as this could provide additional insight into whether HDL functionality reflects complementary biological mechanisms involved in disease progression.

Second, our dataset comes from the PREDIMED study (a nutritional intervention trial) which analyzed as a prospective cohort, while adjusting for intervention group. As these findings are observational, residual confounding cannot be excluded despite comprehensive adjustment for several CVD and dyslipidemia risk factors. Therefore, casual inferences should be made with caution. In addition, given the high cardiovascular risk of this cohort, competing-risk methods could provide complementary information regarding the cumulative incidence of dementia.

Third, some cholesterol-related biomarkers assessed represent ex vivo functional tests using cellular models to approximate HDL functions. While these assays provide valuable physiological insights, they may not fully reflect in vivo dynamics due to the influence of compensatory mechanisms in humans. With this regard, functionality of circulating HDL may not fully recapitulate those of central nervous system-derived HDL particles. In addition, astrocytes are the principal cell type responsible for cholesterol efflux via HDL-like particles [30]. Thus, the evaluation of HDL-mediated cholesterol efflux in neuronal cells may not fully reflect the complex, interconnected mechanisms governing cerebral cholesterol homeostasis, potentially limiting the physiological relevance of the findings. Astrocytes constitute the most physiologically relevant model for investigating cholesterol efflux in the central nervous system, with this regard cholesterol efflux from astrocytes is induced by both lipid-free apolipoproteins and lipoproteins whereas cholesterol efflux from neurons is primarily triggered by lipoproteins [31]. Furthermore, experimental models specifically designed to evaluate the contribution of individual cholesterol transporters and receptors would be of great interest for future investigations.

Finally, the study population consisted of older adults at high cardiovascular risk, which may limit the generalizability of our results to other groups with different baseline risk profiles. The homogeneity of the study population may limit the generalizability of the findings to younger individuals, populations at lower cardiovascular risk, different pathologies, or those from different geographic or ethnic backgrounds. Therefore, replication of these findings in populations with different demographic characteristics, cardiovascular risk profiles, and clinical conditions is warranted.

## 4. Materials and Methods

### 4.1. Study Participants and Design

The present analysis follows a case–cohort design nested within the Prevención con Dieta Mediterránea (PREDIMED) trial. This trial was a multicenter, randomized controlled dietary-intervention study conducted in Spain to evaluate the effects of a traditional Mediterranean diet on primary prevention of CVD. Participants were free of clinical CVD and dementia at baseline yet presented a high CVD risk profile. Eligible participants were men aged 55–80 years or women aged 60–80 years who had type-2 diabetes or met at least three of the following criteria: current smoking, overweight/obesity, hypertension, elevated LDL-C, reduced HDL-C, or a family history of premature CVD [32,33]. The registration is available at http://www.controlled-trials.com/ISRCTN35739639, registered on 5 October 2005 (accessed on 28 September 2026).

For the case–cohort design, we selected 106 incident dementia cases with available biological samples, and a random sub-cohort of 209 participants from the full PREDIMED population who also had available biological samples. Eight dementia cases overlapped with the sub-cohort, yielding a final dataset of 106 dementia cases (82 classified as incident AD) and 201 non-cases of dementia (Figure 1). Within this group, 272 individuals had follow-up samples obtained after one year (71 incident dementia cases [52 of them AD cases] and 201 non-cases). This study is reported following the Strengthening the Reporting of Observational Studies in Epidemiology guidelines [34].

### 4.2. Outcome

The composite dementia outcome comprised AD [35], dementia with Lewy bodies [36], frontotemporal lobar degeneration [37], vascular dementia [38], and normal-pressure hydrocephalus [39]. End-point detection relied on four complementary sources: periodic contact with participants, communications with their primary-care physicians, annual reviews of medical files, and consultation of the National Death Index. Only events that occurred between 1 October 2003 and 30 June 2012 and were confirmed by the Clinical Adjudication Committee of the study were eligible for analysis. Data from three PREDIMED centers were omitted because they lacked a comprehensive search in medical records for dementia diagnoses during follow-up.

### 4.3. Lipid Profile

Serum samples were collected after an overnight fast, shipped to a central laboratory, and stored at −80 °C until analysis. Case and non-case samples were randomized in the analytical series. Routine biochemical measurements were performed in accredited local laboratories with external quality-control standards. Total cholesterol and triglycerides were quantified with the cholesterol esterase–oxidase–peroxidase and glycerol phosphate oxidase–peroxidase assays, respectively. HDL-C was measured after precipitating apolipoprotein B-containing lipoproteins with phosphotungstic acid and magnesium chloride. When triglyceride levels were below 3.39 mmol/L, LDL-C was estimated with the Friedewald equation.

### 4.4. HDL Functionality and Cholesterol-Related Determinations

HDL functionality assays were performed in apolipoprotein B-depleted plasma samples, which are plasma samples in which HDLs were the only lipoproteins present. They were prepared by precipitating apolipoprotein B-containing lipoproteins using 20% polyethylene glycol 8000 (Sigma-Aldrich, Madrid, Spain) [40].

We measured HDL cholesterol efflux capacity from human neurons using a SH-SY5Y neuron-like cell model. SH-SY5Y neuroblastoma cells (American Type Culture Collection CRL-2266™, 10801 University Boulevard, Manassas, VA, USA) were cultured to confluence in the recommended medium, a 1:1 mixture of Eagle’s Minimum Essential Medium and F-12 medium, supplemented with 10% (*v*/*v*) fetal bovine serum, amino acids and penicillin/streptomycin. Cells were then differentiated for 4 days with 10 µM all-trans retinoic acid. After rinsing, cells were loaded for 24 h with 0.025 mM BODIPY fluorescent cholesterol, washed again and equilibrated for 24 h in serum-free medium containing 1% bovine serum albumin. Cholesterol efflux was initiated by adding apolipoprotein-B-depleted plasma to a final concentration of 6.6% (*v*/*v*) and incubating the plates for 16 h. Fluorescence was measured in both cell lysates and culture supernatants at 485 nm excitation/535 nm emission using an Infinite M200 plate reader (Tecan Group Ltd., Männedorf, Switzerland). Blank-corrected values were used to calculate cholesterol efflux capacity as follows: [fluorescence in supernatants/(fluorescence in supernatants + fluorescence in cells)] × 100 [13]. Intra- and inter-assay coefficients of variation in cholesterol efflux capacity from SH-SY5Y neurons were 1.04% and 3.5%, respectively.

We assessed HDL ability to protect brain endothelial cells from inflammatory injury by measuring how effectively they reduced the release of soluble vascular cell adhesion molecule-1 (sVCAM-1) after stimulation with tumor necrosis factor-α in HBEC-5i human brain microvascular endothelial cells (American Type Culture Collection CRL-3245™; University Boulevard, Manassas, VA, USA). HBEC-5i cells were expanded in gelatin-coated T-75 flasks and grown to confluence in a 1:1 mixture of Dulbecco’s Modified Eagle Medium and F-12 medium containing 10% (*v*/*v*) fetal bovine serum, l-glutamine, and penicillin/streptomycin. Monolayers (5 × 10^−4^ cells per well) were incubated with 10% (*v*/*v*) apolipoprotein-B depleted plasma for 24 h. After the incubation, cells were rinsed and tumor necrosis factor-α (10 ng/mL) was added for 24 h. Culture supernatants were collected, and sVCAM-1 was quantified by enzyme-linked immunosorbent assay (ThermoFisher, Thermo Fisher Scientific, Waltham, MA, USA: ref.: KHT0601) [41]. HDL capacity to protect brain endothelial cells was expressed as follows: [1/(supernatant of cells treated with apolipoprotein-B depleted plasma minus sVCAM in the chromogen)/sVCAM-1 in the pool of the supernatant of cells treated with apolipoprotein-B depleted plasma minus sVCAM in the chromogen)] × 100. Intra-assay coefficient of variation in protection from injury of HBEC-5i brain microvascular endothelial cells was 12.37%.

ApoE levels were quantified in plasma using a turbidimetry commercial kit from Spinreact in an ABX Pentra-400 autoanalyzer (Spinreact, S.A.U., Sant Esteve de Bas, Girona, Spain). 24S-HC concentrations were quantified in serum by an enzyme-linked immunosorbent assay (BioVision, BioVision, Inc., Milpitas, CA, USA; ref.: #K4434-100).

### 4.5. Other Variables

Trained health professionals collected initial data on age (continuous), sex, educational attainment (primary/secondary/higher), the prevalence of CVD risk factors at baseline (diabetes, hypercholesterolemia, hypertriglyceridemia, and hypertension: yes/no), body mass index (continuous), and smoking status (never/former/current smoker) [32,33]. Diabetes was defined by fasting glucose levels > 6.99 mmol/L or the use of insulin or glucose-lowering medications. Hypercholesterolemia was defined by total cholesterol levels > 5.17 mmol/L or the use of cholesterol-lowering medications. Hypertriglyceridemia was defined by triglyceride levels > 1.69 mmol/L or the use of triglyceride-lowering medications. Hypertension was defined as systolic blood pressure > 140 mmHg, diastolic blood pressure > 90 mmHg, or the use of antihypertensive medication. We also determined whether the participants carried the ε4 allele in the *APOE* gene.

### 4.6. Sample Size

A case–cohort design with 106 cases and 209 randomly chosen participants from the full cohort of 7447 (including 201 non-cases), assuming a two-sided α error of 5% and a dementia incidence of 1.48% over a median follow-up of 6.1 years, provided ≥80% power to detect a Hazard Ratio (HR) of 1.79 for each one-standard deviation (SD) increase in the cholesterol-related biomarkers. Power calculations were performed using the ccsize function in the R package gap version 4.3.1.

### 4.7. Statistical Analysis

Normally distributed continuous variables were described using means and SDs, while non-normally distributed continuous variables were presented as medians and interquartile ranges (1st–3rd quartiles). Categorical variables were reported as proportions. To assess differences in baseline characteristics between participants with and without dementia onset, we used t-tests for normally distributed continuous variables, Mann–Whitney U-tests for non-normally distributed continuous variables, and chi-squared tests for categorical variables.

Associations between cholesterol related-biomarkers and the risk of incident dementia were evaluated using the Lin-Ying modification of Cox proportional hazards regression models adapted for the case–cohort design [42], implemented using the cch function in the R survival package. The case–cohort sampling design was specified through the subcohort indicator and the size of the original cohort (cohort size = 7447); the Lin–Ying estimator uses this information to account for the subcohort sampling scheme in the estimation of regression coefficients and their variance. Thus, no additional manually specified sampling weights were required, and variance estimates were those provided by the Lin–Ying case–cohort estimator implemented in cch. Results were expressed as HRs per one-SD increment in cholesterol-related biomarker levels. Three models were constructed: unadjusted (model 1); adjusted for age, sex, recruitment site, PREDIMED intervention group, educational level, smoking status, body mass index, APOE-ε4 genotype, hypertension, diabetes, hypercholesterolemia, and hypertriglyceridemia (model 2); and adjusted for age, sex, recruitment site, PREDIMED intervention group, educational level, smoking status, body mass index, APOE-ε4 genotype, hypertension, diabetes, HDL-C, LDL-C, cholesterol-lowering medication, triglycerides, and triglyceride-lowering medication (model 3; unfolding the dyslipidemia covariates in model 2). For the lipid profile measurements (total cholesterol, LDL cholesterol, HDL cholesterol, and triglycerides), only models 1 and 2 are presented. Missing values for variables where they existed (educational level [0.4% of participants], HDL-C [2.2% of participants], LDL-C [4.2% of participants], triglycerides [1.6% of participants]) were imputed using the random forest method [43]. Using the same set of models, we examined the associations between one-year differences in the cholesterol-related biomarkers and dementia incidence (in the subset of individuals with biomarker data after one year of follow-up), as well as the relationships of baseline values and one-year differences in the cholesterol-related biomarkers with the incidence of AD, excluding non-AD dementia cases from these analyses.

Analyses were conducted in R (version 4.3.1). Code for database creation, data management and statistical analysis is available at https://github.com/alvarohernaez/dementia_cohorts (accessed on 28 September 2026).

## 5. Conclusions

In conclusion, HDL functional properties, specifically their capacity to remove excess cholesterol from neurons and to protect brain microvascular endothelial cells, along with lower circulating concentrations of 24S-HC, were associated with a reduced incidence of dementia and AD in older adults at high cardiovascular risk. Given the current lack of effective prognostic biomarkers, our results carry substantial public health relevance, especially considering that AD is the second leading non-communicable cause of death in high-income countries, following ischemic heart disease [44]. Future research should investigate how modifiable lifestyle factors, such as diet and physical activity, and other therapeutic interventions influence these cholesterol-related biomarkers throughout the life course. Further work is also warranted to assess the utility of HDL function-related biomarkers in enhancing dementia and AD prediction across diverse populations and over extended time frames.

## Figures and Tables

**Table 1:** Characteristics of the study population.

	All Participants (*n* = 307)	Dementia Cases (*n* = 106)	Non-Cases (*n* = 201)	*p*-Value
Age, years (mean ± SD)	68.3 (6.46)	71.7 (4.97)	66.6 (6.44)	<0.001
Female sex (*n*, %)	174 (56.7%)	66 (62.3%)	108 (53.7%)	0.189
Primary education (*n*, %)	233 (75.9%)	87 (82.1%)	146 (72.6%)	0.267
Hypertension (*n*, %)	262 (85.3%)	88 (83.0%)	174 (86.6%)	0.505
Type II diabetes (*n*, %)	140 (45.6%)	66 (62.3%)	74 (36.8%)	<0.001
Hypercholesterolemia (*n*, %)	217 (70.7%)	65 (61.3%)	152 (75.6%)	0.013
Hypertriglyceridemia (*n*, %)	89 (29.0%)	39 (36.8%)	50 (24.9%)	0.04
Body mass index categories:				0.451
<25 kg/m^2^ (*n*, %)	29 (9.45%) (85.3%)	13 (12.3%)	16 (7.96%)	
25.0–29.9 kg/m^2^ (*n*, %)	134 (43.6%)	46 (43.4%)	88 (43.8%)	
≥30 kg/m^2^ (*n*, %)	144 (46.9%)	47 (44.3%)	97 (48.3%)	
Tobacco use:				0.04
Never smokers (*n*, %)	188 (61.2%)	73 (68.9%)	115 (57.2%)	
Current smokers (*n*, %)	35 (11.4%)	6 (5.66%)	29 (14.4%)	
Former smokers (*n*, %)	84 (27.4%)	27 (25.5%)	57 (28.4%)	

## Data Availability

Code for database creation, data management and statistical analysis is available at https://github.com/alvarohernaez/dementia_cohorts (accessed on 28 September 2026). Due to national data-protection laws and ethical requirements limiting public release (participants did not give explicit written consent for open sharing of their data), the dataset from this study cannot be made publicly accessible. Nonetheless, de-identified data used in the manuscript can be shared with qualified researchers for collaborative projects. Interested investigators should submit a written request to the PREDIMED Steering Committee at predimed-steering-committee@googlegroups.com.

## Supplementary Materials

The following supporting information can be downloaded at https://www.mdpi.com/article/10.3390/ijms27209197/s1.

## Abbreviations

The following abbreviations are used in this manuscript:

24S-HC	24S-hydroxycholesterol
AD	Alzheimer’s disease
ApoE	apolipoprotein E
BBB	blood–brain barrier
CVD	cardiovascular disease
HDL	high-density lipoprotein
HDL-C	high-density lipoprotein cholesterol
HR	hazard ratio
LDL-C	low-density lipoprotein cholesterol
Predimed	Prevención con Dieta Mediterránea
SD	standard deviation
sVCAM-1	soluble vascular cell adhesion molecule-1
