# Supplementary material for: HDL Functionality, Cholesterol-Related Biomarkers and Incidence of Dementia: A Case–Cohort Study in Older Adults at High Cardiovascular Risk
Source: Int J Mol Sci. 2026 Oct 16;27(20):9197. doi: 10.3390/ijms27209197 (PMC13705062; doi:10.3390/ijms27209197)
Supplement: Supplementary file 1 [file ijms-27-09197-s001.zip › ijms-4459150-supplementary.pdf]

**Supplementary Materials**

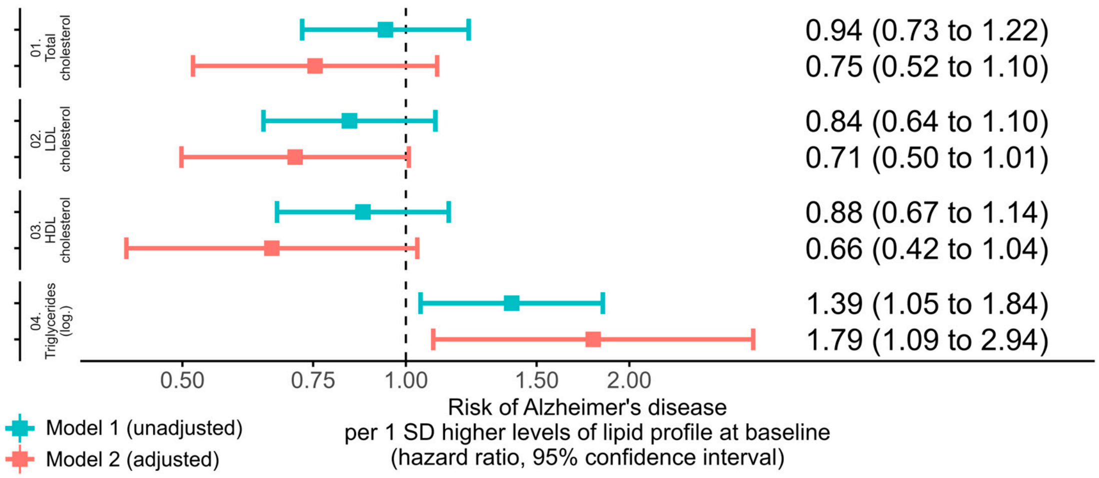


**Supplementary Figure S1**. Association of baseline values of lipid profile with incidence of AD. Model 1: unadjusted. Model 2: adjusted for age, sex, recruitment site, PREDIMED intervention group, educational level, smoking status, body mass index, apoE-ε4 genotype, hypertension, diabetes, hypercholesterolemia, and hypertriglyceridemia.

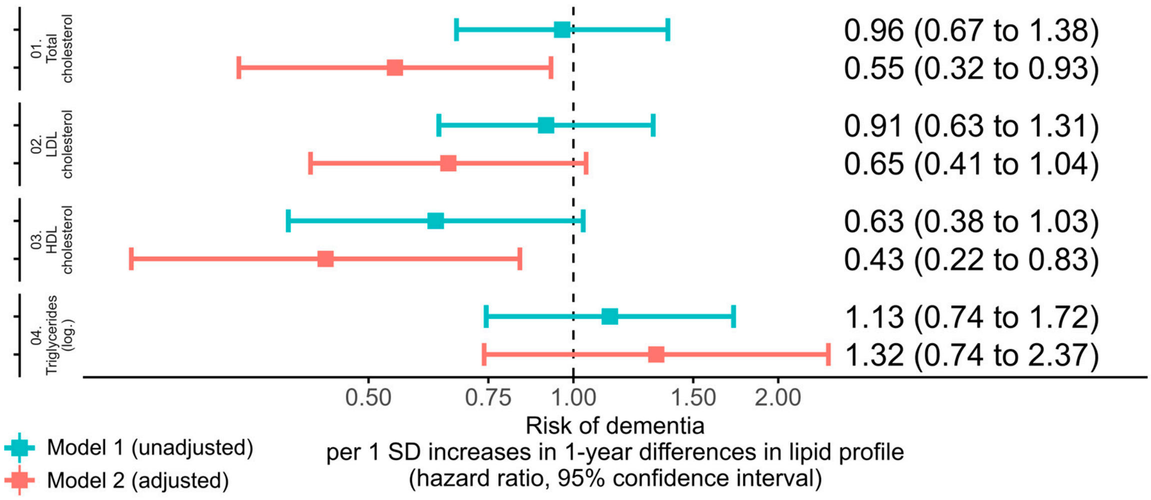


**Supplementary Figure S2**. Association of one-year changes in lipid profile with incidence of dementia. Model 1: unadjusted. Model 2: adjusted for age, sex, recruitment site, PREDIMED intervention group, educational level, smoking status, body mass index, apoE-ε4 genotype, hypertension, diabetes, hypercholesterolemia, and hypertriglyceridemia.

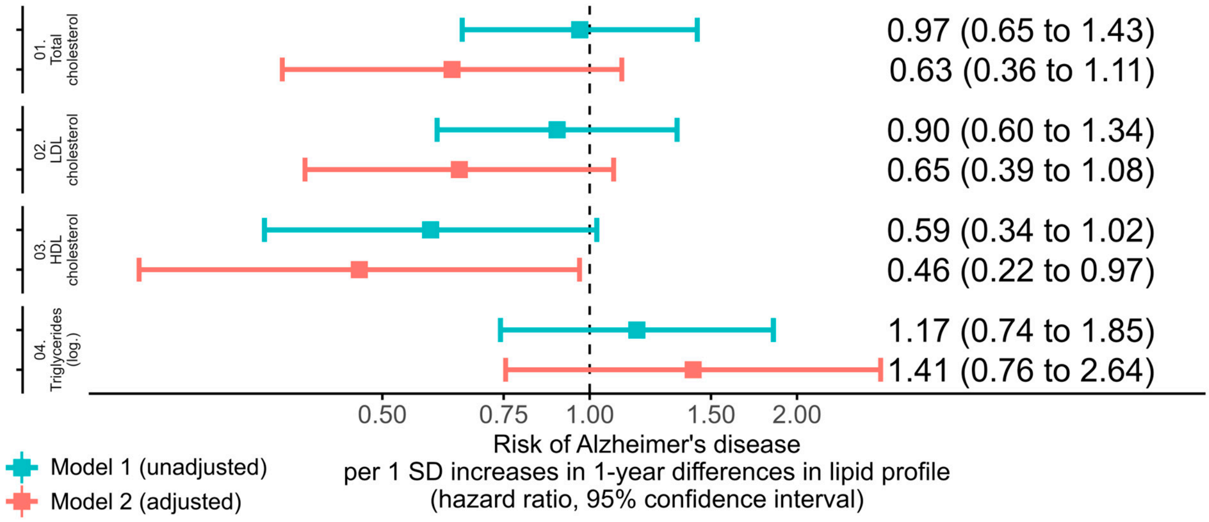


**Supplementary Figure S3**. Association of one-year changes in lipid profile with incidence of AD. Model 1: unadjusted. Model 2: adjusted for age, sex, recruitment site, PREDIMED intervention group, educational level, smoking status, body mass index, apoE-ε4 genotype, hypertension, diabetes, hypercholesterolemia, and hypertriglyceridemia.

**Supplementary Table S1.** Comparison of AD cases with non-cases.

| | AD cases ($n$ = 82) | Non-cases ($n$ = 201) | $p$-value |
|---|---|---|---|
| Age, years (mean ± SD) | 71.9 (4.99) | 66.6 (6.44) | <0.001 |
| Female sex ($n$, %) | 56 (68.3%) | 108 (53.7%) | 0.034 |
| Primary education ($n$, %) | 68 (82.9%) | 146 (72.6%) | 0.320 |
| Hypertension ($n$, %) | 67 (81.7%) | 174 (86.6%) | 0.390 |
| Type II diabetes ($n$, %) | 48 (58.5%) | 74 (36.8%) | 0.001 |
| Hypercholesterolemia ($n$, %) | 51 (62.2%) | 152 (75.6%) | 0.033 |
| Hypertriglyceridemia ($n$, %) | 27 (32.9%) | 50 (24.9%) | 0.217 |
| Body mass index categories: | | | 0.534 |
| <25 kg/m$^2$ ($n$, %) | 10 (12.2%) | 16 (7.96%) | |
| 25.0-29.9 kg/m$^2$ ($n$, %) | 34 (41.5%) | 88 (43.8%) | |
| ≥30 kg/m$^2$ ($n$, %) | 38 (46.3%) | 97 (48.3%) | |
| Tobacco use: | | | 0.009 |
| Never smokers ($n$, %) | 62 (75.6%) | 115 (57.2%) | |
| Current smokers ($n$, %) | 4 (4.88%) | 29 (14.4%) | |
| Former smokers ($n$, %) | 16 (19.5%) | 57 (28.4%) | |

**Supplementary Table S2.** Comparison of the Non-AD Dementia Subgroup with the AD Subgroup.

| | Non-AD dementia | Alzheimer's disease | P-value |
|---|---|---|---|
| Age, years (mean ± SD) | 71.2 (4.97) | 71.9 (4.99) | 0.558 |
| Female sex (*n*, %) | 10 (41.7%) | 56 (68.3%) | 0.033 |
| PREDIMED intervention group (*n*, %) | | | 0.627 |
| Extravirgin olive oil | 7 (29.2%) | 32 (39.0%) | |
| Nuts | 7 (29.2%) | 18 (22.0%) | |
| Low-fat diet control group | 10 (41.7%) | 32 (39.0%) | |
| Education level (n, %) | | | 0.731 |
| Primary (n, %) | 19 (79.2%) | 68 (82.9%) | |
| Secondary (n, %) | 3 (12.5%) | 9 (11.0%) | |
| University (n, %) | 2 (8.33%) | 5 (6.10%) | |
| Type II diabetes (*n*, %) | 18 (75.0%) | 48 (58.5%) | 0.221 |
| Hypercholesterolemia (*n*, %) | 14 (58.3%) | 51 (62.2%) | 0.918 |
| Hypertriglyceridemia (*n*, %) | 12 (50.0%) | 27 (32.9%) | 0.199 |
| Hypertension (*n*, %) | 21 (87.5%) | 67 (81.7%) | 0.758 |
| Body mass index categories: | | | 0.725 |
| <25 kg/m$^2$ (*n*, %) | 3 (12.5%) | 10 (12.2%) | |
| 25.0-29.9 kg/m$^2$ (*n*, %) | 12 (50.0%) | 34 (41.5%) | |
| ≥30 kg/m$^2$ (*n*, %) | 9 (37.5%) | 38 (46.3%) | |
| Tobacco use: | | | |
| Never smokers (*n*, %) | 11 (45.8%) | 62 (75.6%) | 0.016 |
| Current smokers (*n*, %) | 2 (8.33%) | 4 (4.88%) | NA |
| Former smokers (*n*, %) | 11 (45.8%) | 16 (19.5%) | NA |

NA, non available.

**Supplementary Table S3**. Analyses of the Non-AD Dementia Subgroup in the Case Cohort Design.

| Per +1 SD: | HR (model 1) | p value (model 1) | HR (model 2) | p value (model 2) | HR (model 3) | p value (model 3) |
|---|---|---|---|---|---|---|
| Cholesterol efflux (SH-SY5Y cells) | 1.20 (0.77 to 1.87) | 0.421 | 1.19 (0.68 to 2.07) | 0.539 | 1.24 (0.69 to 2.23) | 0.48 |
| VCAM1 release (HBEC-5i cells) | 1.05 (0.63 to 1.73) | 0.852 | 1.16 (0.63 to 2.15) | 0.635 | 1.08 (0.56 to 2.08) | 0.81 |
| 24S-hydroxycholesterol | 1.00 (0.62 to 1.60) | 0.987 | 0.97 (0.53 to 1.77) | 0.917 | 1.06 (0.56 to 1.98) | 0.861 |
| Circulating ApoE | 0.85 (0.51 to 1.41) | 0.533 | 0.75 (0.40 to 1.40) | 0.367 | 0.32 (0.12 to 0.83) | 0.02 |
| Total cholesterol | 0.99 (0.65 to 1.50) | 0.961 | 1.01 (0.62 to 1.64) | 0.973 | 10.5 (0.018 to 6108) | 0.469 |
| LDL-cholesterol | 1.00 (0.66 to 1.51) | 0.995 | 1.09 (0.69 to 1.72) | 0.725 | 1.35 (0.80 to 2.27) | 0.268 |
| HDL-cholesterol | 0.52 (0.31 to 0.88) | 0.015 | 0.51 (0.26 to 1.00) | 0.051 | 0.38 (0.17 to 0.84) | 0.017 |
| Triglycerides (logarithm) | 1.58 (1.01 to 2.47) | 0.047 | 1.44 (0.62 to 3.35) | 0.394 | 2.52 (0.37 to 17.1) | 0.345 |

**Supplementary Table S4**. Comparison of the absolute values at baseline and at the one-year follow-up.

| Variable | Population | N | Baseline, mean (SD) | 1 year, mean (SD) | Mean change (95% CI) | P value |
|---|---|---|---|---|---|---|
| Cholesterol efflux | Whole population | 269 | 0.96 (0.1) | 0.94 (0.1) | -0.02 (-0.03 to -0.01) | <0.001 |
| Cholesterol efflux | Incident dementia | 68 | 0.94 (0.09) | 0.91 (0.1) | -0.03 (-0.06 to -0.01) | 0.008 |
| Cholesterol efflux | No dementia | 201 | 0.97 (0.1) | 0.95 (0.1) | -0.02 (-0.03 to -0.01) | 0.003 |
| Endothelial protection | Whole population | 269 | 31.66 (7.6) | 32.68 (6.55) | 1.02 (-0.01 to 2.05) | 0.051 |
| Endothelial protection | Incident dementia | 68 | 30.25 (8.92) | 31.26 (7.69) | 1.01 (-1.46 to 3.49) | 0.417 |
| Endothelial protection | No dementia | 201 | 32.14 (7.05) | 33.16 (6.06) | 1.02 (-0.08 to 2.13) | 0.069 |
| 24S-hydroxycholesterol | Whole population | 240 | 61.76 (11.17) | 62.02 (10.46) | 0.26 (-0.88 to 1.41) | 0.653 |
| 24S-hydroxycholesterol | Incident dementia | 57 | 63.47 (12.66) | 61.9 (10.81) | -1.57 (-4.6 to 1.45) | 0.302 |
| 24S-hydroxycholesterol | No dementia | 183 | 61.23 (10.65) | 62.06 (10.38) | 0.83 (-0.35 to 2.01) | 0.165 |
| Apolipoprotein E | Whole population | 263 | 5.01 (1.43) | 4.86 (1.4) | -0.14 (-0.28 to -0.01) | 0.034 |
| Apolipoprotein E | Incident dementia | 63 | 4.99 (1.63) | 4.69 (1.53) | -0.31 (-0.65 to 0.04) | 0.085 |
| Apolipoprotein E | No dementia | 200 | 5.01 (1.37) | 4.92 (1.35) | -0.09 (-0.23 to 0.04) | 0.181 |
| Total cholesterol | Whole population | 263 | 209.73 (36.89) | 203.42 (33.77) | -6.31 (-9.96 to -2.66) | <0.001 |
| Total cholesterol | Incident dementia | 77 | 211.58 (34.36) | 205.43 (34.32) | -6.16 (-12.08 to -0.24) | 0.042 |
| Total cholesterol | No dementia | 186 | 208.97 (37.96) | 202.59 (33.61) | -6.37 (-10.95 to -1.8) | 0.007 |
| LDL-cholesterol | Whole population | 249 | 132.89 (32.71) | 128.2 (30.29) | -4.69 (-8.11 to -1.28) | 0.007 |
| LDL-cholesterol | Incident dementia | 72 | 132.94 (29.37) | 128.38 (31.66) | -4.56 (-10.26 to 1.15) | 0.116 |
| LDL-cholesterol | No dementia | 177 | 132.87 (34.06) | 128.13 (29.81) | -4.75 (-8.99 to -0.51) | 0.028 |
| HDL-cholesterol | Whole population | 257 | 52.33 (11.06) | 52.38 (10.78) | 0.04 (-0.83 to 0.92) | 0.925 |
| HDL-cholesterol | Incident dementia | 76 | 50.66 (10.32) | 51.65 (9.38) | 0.99 (-0.52 to 2.5) | 0.195 |
| HDL-cholesterol | No dementia | 181 | 53.04 (11.31) | 52.68 (11.33) | -0.36 (-1.43 to 0.72) | 0.513 |

**Supplementary Table S5**. Stratified Analysis by APOE Genotype.

| Variable | APOE4 stratum | N | Cases | HR per 1-SD (model 2) | Pvalue | HR per 1-SD (model 3) | Pvalue |
|---|---|---|---|---|---|---|---|
| Cholesterol efflux | APOE4-negative | 223 | 62 | 0.79 (0.59-1.04) | 0.093 | 0.82 (0.62-1.09) | 0.167 |
| Cholesterol efflux | APOE4-positive | 69 | 38 | 0.83 (0.52-1.32) | 0.432 | 0.69 (0.40-1.20) | 0.188 |
| VCAM1 release | APOE4-negative | 223 | 62 | 0.89 (0.67-1.17) | 0.396 | 0.88 (0.66-1.16) | 0.356 |
| VCAM1 release | APOE4-positive | 69 | 38 | 0.67 (0.48-0.95) | 0.026 | 0.62 (0.42-0.92) | 0.016 |
| 24S-hydroxycholesterol | APOE4-negative | 206 | 56 | 1.13 (0.85-1.49) | 0.401 | 1.15 (0.85-1.57) | 0.355 |
| 24S-hydroxycholesterol | APOE4-positive | 66 | 35 | 0.93 (0.62-1.40) | 0.729 | 0.85 (0.54-1.33) | 0.478 |
| Apolipoprotein E | APOE4-negative | 220 | 60 | 0.97 (0.69-1.36) | 0.846 | 0.89 (0.60-1.32) | 0.567 |
| Apolipoprotein E | APOE4-positive | 67 | 36 | 0.99 (0.58-1.67) | 0.959 | 1.44 (0.74-2.82) | 0.280 |
| Total cholesterol | APOE4-negative | 225 | 64 | 0.97 (0.74-1.27) | 0.828 | 1.98 (0.84-4.71) | 0.120 |
| Total cholesterol | APOE4-positive | 69 | 38 | 0.54 (0.30-0.96) | 0.035 | 3.48 (1.03-11.71) | 0.044 |
| LDL-cholesterol | APOE4-negative | 225 | 64 | 0.98 (0.76-1.26) | 0.870 | 1.01 (0.77-1.32) | 0.966 |
| LDL-cholesterol | APOE4-positive | 69 | 38 | 0.48 (0.28-0.85) | 0.011 | 0.52 (0.31-0.88) | 0.015 |
| HDL cholesterol | APOE4-negative | 225 | 64 | 0.81 (0.59-1.11) | 0.187 | 0.83 (0.60-1.16) | 0.286 |
| HDL-cholesterol | APOE4-positive | 69 | 38 | 1.25 (0.65-2.38) | 0.507 | 1.10 (0.61-1.99) | 0.755 |
| Triglycerides | APOE4-negative | 225 | 64 | 1.36 (0.87-2.14) | 0.178 | 1.22 (0.40-3.78) | 0.727 |
| Triglycerides | APOE4-positive | 69 | 38 | 1.05 (0.59-1.87) | 0.864 | 1.75 (0.45-6.79) | 0.417 |

**Supplementary Table S6**. Comparison of baseline demographics, lipid profiles, and dementia risk factors between participants retained in the study and those lost to follow-up.

| | All<br>N=307 | Not retained<br>N=23 | Retained<br>N=284 | overall p value |
|---|---|---|---|---|
| age | 68.3 (6.46) | 72.7 (5.67) | 68.0 (6.39) | 0.001 |
| sex | | | | 0.839 |
| Male | 133 (43.3%) | 9 (39.1%) | 124 (43.7%) | |
| Female | 174 (56.7%) | 14 (60.9%) | 160 (56.3%) | |
| Intervention group | | | | <0.001 |
| Extravirgin olive oil | 116 (37.8%) | 2 (8.70%) | 114 (40.1%) | |
| Nuts | 95 (30.9%) | 5 (21.7%) | 90 (31.7%) | |
| Low-fat diet control group | 96 (31.3%) | 16 (69.6%) | 80 (28.2%) | |
| Education level: | | | | 0.845 |
| Primary | 233 (75.9%) | 19 (82.6%) | 214 (75.4%) | |
| Secondary | 44 (14.3%) | 2 (8.70%) | 42 (14.8%) | |
| University | 27 (8.79%) | 2 (8.70%) | 25 (8.80%) | |
| Type II diabetes (*n*, %) | | | | <0.001 |
| No | 167 (54.4%) | 3 (13.0%) | 164 (57.7%) | |
| Yes | 140 (45.6%) | 20 (87.0%) | 120 (42.3%) | |
| Hypercholesterolemia (*n*, %) | | | | 0.189 |
| No | 90 (29.3%) | 10 (43.5%) | 80 (28.2%) | |
| Yes | 217 (70.7%) | 13 (56.5%) | 204 (71.8%) | |
| Hypertriglyceridemia (*n*, %) | | | | 1.000 |
| No | 218 (71.0%) | 16 (69.6%) | 202 (71.1%) | |
| Yes | 89 (29.0%) | 7 (30.4%) | 82 (28.9%) | |
| Hypertension (*n*, %) | | | | 0.354 |
| No | 45 (14.7%) | 5 (21.7%) | 40 (14.1%) | |
| Yes | 262 (85.3%) | 18 (78.3%) | 244 (85.9%) | |
| Body mass index categories: | | | | 0.138 |
| <25 kg/m$^2$ (*n*, %) | 29 (9.45%) | 5 (21.7%) | 24 (8.45%) | |
| 25.0-29.9 kg/m$^2$ (*n*, %) | 134 (43.6%) | 9 (39.1%) | 125 (44.0%) | |
| ≥30 kg/m$^2$ (*n*, %) | 144 (46.9%) | 9 (39.1%) | 135 (47.5%) | |
| Tobacco use: | | | | 0.301 |
| Never smokers (*n*, %) | 188 (61.2%) | 18 (78.3%) | 170 (59.9%) | |
| Current smokers (*n*, %) | 35 (11.4%) | 1 (4.35%) | 34 (12.0%) | |
| Former smokers (*n*, %) | 84 (27.4%) | 4 (17.4%) | 80 (28.2%) | |
